# Treatment Outcomes of Rare Retromolar Trigone Squamous Cell Carcinoma Using Combined Modalities

**DOI:** 10.7759/cureus.1203

**Published:** 2017-05-01

**Authors:** Muhammad Faisal, Taskheer Abbas, Usman Khaleeq, Mohammad Adeel, Abdul Wahid Anwer, Raza Hussain, Arif Jamshed

**Affiliations:** 1 Department of Surgical Oncology, Shaukat Khanum Memorial Cancer Hospital and Research Center, Lahore, Pakistan; 2 Radiation Oncology, Shaukat Khanum Memorial Cancer Hospital and Research Center, Lahore, Pakistan

**Keywords:** retromolar trigone, squamous cell carcinoma, oral cavity tumors

## Abstract

**Background:**

Retromolar trigone squamous cell carcinoma is relatively uncommon and due to its complex anatomy has always remained a challenge in terms of loco-regional control and survival. Surgery, radiotherapy, and chemotherapy as combined modalities have been used but high recurrence rates result in poor outcome.

**Methods:**

We have retrospectively evaluated records of 62 patients treated in Head and Neck Oncology unit of Shaukat Khanum Memorial Cancer Hospital and Research Centre (SKMCH and RC), Lahore, Pakistan from 2004 to 2014 who were included based on the criteria of histopathological proven squamous cell carcinoma of retromolar trigone (RMT) treated with radical intent. Diagnostic workup for all patients involved clinical examination, imaging modalities usually magnetic resonance imaging (MRI), computerized tomography (CT), Orthopantomogram (OPG), and chest x-ray (CXR) to evaluate regional and distant metastasis, respectively. Kaplan-Meier survival curves were used to depict survival.

**Results:**

The study was comprised of 36 male and 26 female patients. Treatment modalities used are surgery only (n = 1), radiotherapy alone (n = 13), radiotherapy followed by surgery (n = 10), chemoradiotherapy (n = 16), induction chemotherapy followed by concurrent chemoradiotherapy (n = 19), induction chemotherapy followed by surgery, and radiotherapy (n = 2). Surgical interventions include wide local excisions (n = 6), marginal mandibulectomy (n = 4), and segmental mandibulectomy (n = 4). Surgical margins were clear in 54%, close in 38%, and involved in 8% of patients. AJCC 7th edition showed cT1 8%, cT2 22%, cT3 14%, and cT4 56% while pT1 2%, pT2 3%, and pT4 8%. During follow-up, 18% patients have come up with local recurrence, 22% showed persistent disease while 9% have presented with distant metastasis. The five-year and overall survivals are 38% and 22%, respectively.

**Conclusion:**

Retromolar trigone involvement poses many vital structures at risk of involvement. Late presentation results in involvement of masticator space compromising both mouth opening and surgical outcomes. Surgery and radiotherapy have shown comparable results in disease control. Bone invasion has shown poor outcome in terms of loco-regional control and overall survival.

## Introduction

The retromolar trigone (RMT) in the dry mandible is a triangular area bounded by temporal crest on the medial side, anterior border of ramus on the lateral side, and base posterior to the socket for the third molar [1]. Although RMT tumors are uncommon as compared to other oral sub-sites, squamous cell carcinoma is the most common histopathological presentation. Late stage presentation due to the absence of early symptoms and involvement of adjacent sub-sites such as buccal mucosa, anterior faucial pillar, upper and lower alveolus are the characteristic features of RMT squamous cell carcinoma. Bone invasion has resulted in more morbidity and poor outcomes [2-4]. Pain and trismus are delayed symptoms secondary to involvement of adjacent nerves and muscles of mastication [5]. Little has been published regarding the clear guidelines in terms of management with surgery, radiotherapy, and chemoradiation being used either as single modality or in combination. The objective of our study is to share our experience as the only tertiary care high volume center in a developing country with retromolar trigone squamous cell carcinoma for the clinicopathological outcomes, patterns of failure, and the impact of different treatment modalities on survival.

## Materials and methods

We have retrospectively evaluated records of 66 patients treated in Head and Neck Oncology unit of Shaukat Khanum Memorial Cancer Hospital and Research Centre (SKMCH and RC), Lahore, Pakistan from January, 2004 to December, 2014 who were included in the study based on the criteria of histopathological and radiological diagnosis of squamous cell carcinoma of RMT. As per protocol, exemption from Institutional Review Board of SKMCH and RC has been granted due to retrospective nature of the study. Diagnostic workup including computerized tomography/magnetic resonance imaging (CT/MRI), Orthopantomogram (OPG), and chest x-ray (CXR) was done. All these patients were discussed in MDT before any intervention. Tumors were staged based on TNM system by AJCC 7th edition. Outcomes were measured in terms of overall survival, treatment-related survival, impact of early versus advanced stage on survival, and modes of failure (local, regional or distant).

Treatment was guided based on the extent of the disease, medical condition, and patient’s preference. Patients with mucosal disease with no bony involvement were treated with surgery, surgery followed by radiotherapy based on adverse features such as close or positive margin, perineural invasion, and nodal involvement. Patients with locally advanced disease and severe limitation in mouth opening secondary to trismus related to sub mucous fibrosis or involvement of muscles of mastication, underwent radiotherapy with surgery reserved for persistent disease only and neoadjuvant chemotherapy followed by chemoradiotherapy. Those with obvious bony involvement as indicated in CT scan and adequate mouth opening (more than 30 mm) were managed by marginal/segmental mandibulectomy and neck dissection.

### Statistical analysis

The data were analyzed using IBM SPSS Statistics version 20 (IBM Corp., Armonk, NY, USA). Kaplan–Meier survival curves were used to assess survival outcomes. Variables related to patients such as age, gender, tumor grade, treatment modalities used, clinical and pathological stage and pattern of failure were evaluated and collected.

## Results

There were 36 male and 26 female patients. The median age at diagnosis was 53 years (Range 18-79 years). The median follow-up was 15 months and median time to recurrence was seven months. Histologically, 27 (43%) patients were well differentiated, 29 (47%) were moderately differentiated, and six (11%) were poorly differentiated. Geographical distribution shows most of the patients were from the province of Punjab (n = 49) followed by KPK (n = 9), Sindh (n = 1), and Balouchistan (n = 3). Risk factors were smoking (35%), betel quid (30%), naswar (16%), and alcohol 3% (Table 1).

**Figure 1:**
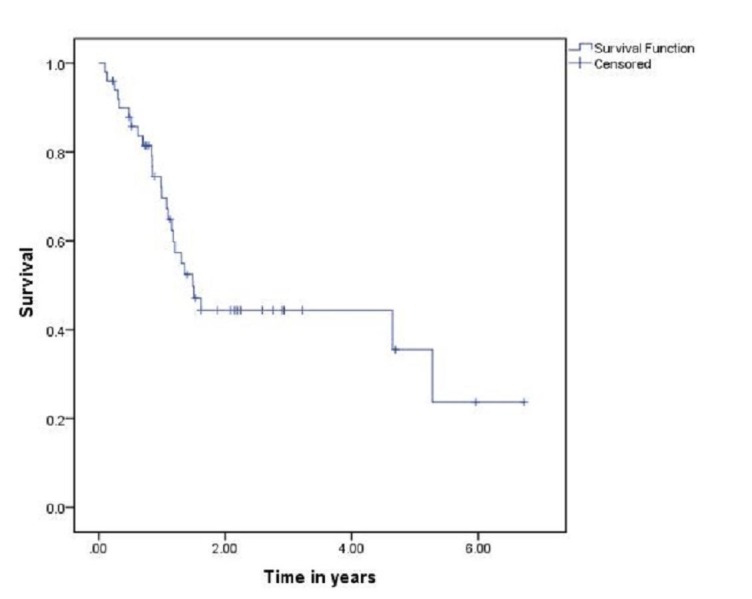
Overall survival

**Figure 2:**
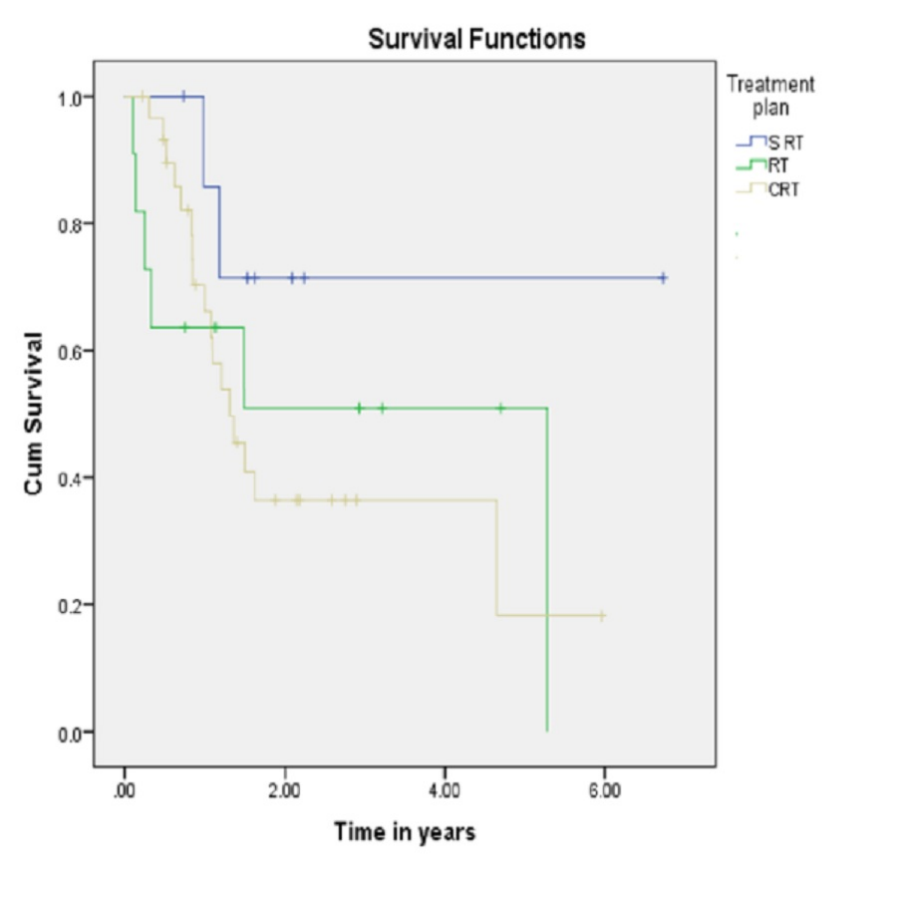
Treatment modality related survival CRT: Chemoradiotherapy; RT: Radiotherapy; S RT: Surgery followed by radiotherapy.

Treatment modalities used were surgery only (n = 1), radiotherapy alone (n = 13), radiotherapy followed by surgery (n = 10), chemoradiotherapy (n = 11), induction chemotherapy followed by radiotherapy (n = 5) induction chemotherapy followed by concurrent chemoradiotherapy (n = 19), induction chemotherapy followed by surgery and radiotherapy (n = 3). Surgical interventions included wide local excisions (n = 6), marginal mandibulectomy (n = 4) and segmental mandibulectomy (n = 4). Out of these, one patient had local failure in segmental mandibulectomy group while marginal mandibulectomy group had two local and one regional failures. Ipsilateral neck dissection was performed as part of ablative resections and showed high occult metastatic rate (62.5%). AJCC 7th edition showed cT1 8%, cT2 22%, cT3 14%, and cT4 56% while pT1 3%, pT2 3%, and pT4 9%. During follow-up, 20% patients have come up with local recurrence, 24% showed persistent disease while 10% have presented with distant metastasis. The five-year and overall survivals are 38% and 22%, respectively (Figure 1). The three-year survival for patients treated with surgery followed by radiotherapy, radiotherapy only, and chemoradiotherapy are 76%, 66%, and 38% respectively (Figure 2). Further, classifying the patients into early (stage 1 and 2) and advanced (stage 3 and 4) disease, improved survival (75%) has been reported in advanced RMT tumors as compared to early ones (32%) (Table 1).

**Table 1:** Demographics and clinicopathological features LVI: Lymphovascular invasion; PNI: Perineural invasion.

Characteristics	N (%)
Age	
<40 yrs	11 (17.7%)
>40 yrs	51 (80.6%)
Sex	
Male	36 (58.1%)
Female	26 (41.9%)
Risk factors	
Smoking	
Yes	22 (35.5%)
No	42 (64.5%)
Betel Nut	
Yes	19 (30.6%)
No	43 (69.4%)
Naswar	
Yes	10 (16.1%)
No	52 (83.9%)
Alcohol	
Yes	02 (3.2%)
No	10 (80%)
Grade	
Well	27 (43.5%)
Moderate	29 (46.8%)
Poor	06 (9.7%)
PNI	
Yes	02 (20%)
No	10 (80%)
LVI	
Yes	01 (10%)
No	06 (90%)
Recurrence pattern	
Local	12 (19.4%)
Regional	06 (9.6%)
Treatment Modality	
Surgery	01 (1.6%)
Surgery + Radiotherapy	10 (16.1%)
Radiotherapy	13 (20.9%)
Chemoradiotherapy	16 (25.8%)
Induction chemo + surgery + radiotherapy	03 (4.8%)
Induction chemo + Concurrent chemo radiotherapy	19 (30.6%)

## Discussion

Oral cancers are among the 10 most common causes of cancer-related deaths in the world. The etiology appears to be multifactorial and strongly related to lifestyle, mostly habits, and diet (particularly tobacco alone or in betel, and alcohol use), although other factors as immune defects or immunosuppression, defects of carcinogen metabolism, or defects in DNA-repair enzymes may be implicated. Human papillomavirus (HPV) infection has also been connected with malignant tumors of the oropharyngeal – retromolar trigone junction, together with the other traditional risk factors [6]. PubMed search with the filter “Retromolar trigone, Squamous cell carcinoma” has yielded 112 results which were further filtered out to include 25 articles strictly relating to Retromolar trigone. Retromolar trigone tumors are rare but more aggressive with poor outcome. There is less published data and mostly is retrospective in nature due to rarity of RMT tumors. The peculiar feature of RMT tumors is close proximity to surrounding structures, early involvement of adjacent sub-sites and underlying bone and significant functional compromise post-surgery and radiotherapy [7]. Tumors involving RMT can extend to involve muscles of mastication, soft palate, tonsillar pillars, floor of mouth, and parapharyngeal space. Clinical examination needs to be supplemented by imaging to figure out the sub-clinical spread of the disease. Contrast-enhanced CT has shown high accuracy for detection of mandibular invasion [8-9]. Both CT and MRI have their indications in RMT tumors with MRI having better detailing of soft tissue extension, masticator space involvement, perineural spread, and infratemporal fossa invasion.

For early lesion with soft tissue involvement sparing bone, wide local excision seems a reasonable option but this is not a usual happening in these tumors. Bone involvement mandates bony resection either marginal or segmental depending on the extent of the disease.

We have performed wide local resections for early disease with no clinical or radiological bone involvement, marginal and segmental mandibulectomy depending on the extent of bone invasion and ipsilateral neck dissections for all. Literature review has shown comparisons between single versus multimodality treatment protocols.

Barbosa in 1959 has reported the ‘retromolar operation’ comprising mandibulectomy with enbloc removal of pterygoids and masseter along with ipsilateral neck dissection. A comparison has also been made between surgery and radiotherapy but a small number of patients and evolution of radiation techniques had questioned the final verdict of this study [10]. Kowalski, et al. have analyzed 114 patients with RMT over 31 years with five-year disease-free and overall survival of 48.9% and 55.3%. He suggested a more extensive approach towards resection and radiotherapy for advanced stage disease and adverse features such as perineural invasion (PNI)/Lymphovascular invasion (LVI).

Pascoal, et al. [11] compared 20 patients undergoing marginal mandibulectomy with 22 treated with segmental mandibulectomy resulting in a failure rate of 35% and 36.4%, respectively. The survival rate in the group treated with marginal mandibulectomy was 55% and for group managed with segmental mandibulectomy was 45% showing no significant difference in terms of local recurrences and survival. We have treated one patient with surgery only showing no loco-regional recurrence, 10 patients with surgery followed by radiotherapy with four patients having local recurrences and one with regional failure. Out of eight patients undergoing mandibulectomy, four patients had marginal mandibulectomy with two local and one regional failure while four had segmental mandibulectomy with only one local failure. Ipsilateral neck dissection has been incorporated as a part of the procedure showing occult metastasis in 62.5% of these patients involving multiple levels of nodes.

Ayad, et al. have reported the use of radiotherapy for small RMT tumors with curative intent in their series of 46 patients advocating its use in advanced stages without bone invasion [12]. Deo, et al. have emphasized on the use of aggressive surgical approach followed by radiotherapy for better outcome rather than using radiotherapy alone [13]. Comparable outcome has been documented by Hitchcock, et al. using surgery followed by RT versus RT alone but more complications related to the former group [14]. Huang, et al. have reviewed 65 patients with a survival rate of 96%, 45%, and 31 % with surgery only, surgery and post-operative radiation, and radiation only respectively during five-year follow-up [15]. In our experience, three-year survival of patients treated with surgery and radiotherapy was 76% as compared to those treated with RT only which is 66%. Among those managed by radiotherapy only, four patients showed persistent disease and two had regional failures.

Various phase III studies and recent meta-analysis have advocated concurrent chemotherapy and RT for advanced stage III–IV head and neck carcinoma [16-18]. Scher, et al. have demonstrated 37% five-year loco-regional control and 70% freedom from distant metastasis in their study on a group of 73 RMT tumor patients [19]. Our results have shown 19 patients treated with induction chemotherapy followed by radiotherapy with a three-year survival of 39%. Out of these, four patients had local failure and six had persistent disease. Concurrent chemo-radiotherapy was offered to five patients with a three-year survival of 38% (Figure 2).

The three-year survival of surgically treated patients with clear, close, and involved margin was 100%, 63%, and 23%, respectively. Binahmed, et al. have shared their experience of RMT tumors in 88 patients with the survival of 68%, 83%, and 0% for clear, close, and involved margins [20].

Lymph nodal involvement is a poor prognostic factor in these patients with previous studies reporting nodal involvement to be between 26 and 80% [21]. Most of the oral cavity tumors have a predictable pattern of drainage to level I-III more commonly involving level IB, RMT tumors usually involve level II and occasionally peri-parotid and retropharyngeal nodes.

**Figure 3:**
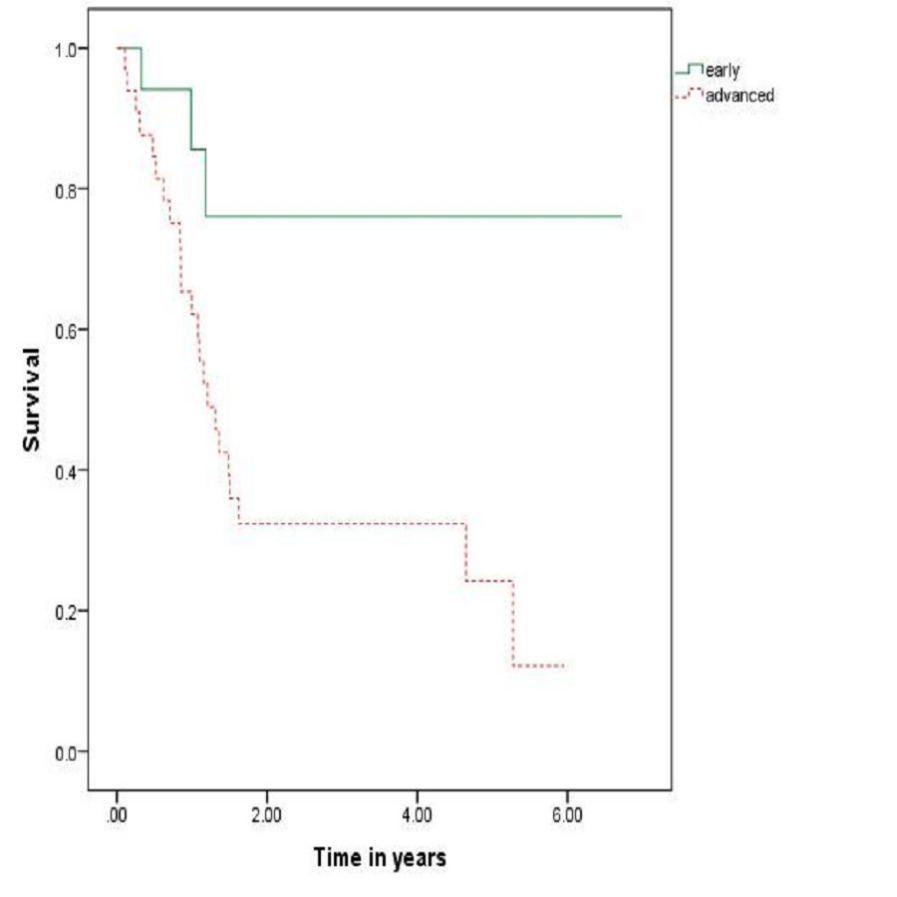
Survival related to early and advanced disease

Currently, no consensus has been developed as far as the treatment guidelines are concerned. Previous studies have certain limitations such as retrospective nature, limited number of patients due to rarity of the disease. Mendenhall, et al. reported five-year overall survivals of 40% and 56% in 35 patients managed with radiation alone and 64 managed with surgery and radiotherapy, respectively [22]. Common practice involves treatment of small tumors (stages I and II) with radiotherapy or surgery and larger tumors (stages III and IV) with combination therapy or surgery alone [23-24]. Early stage disease (stage 1 and 2) has shown improved survival when compared with advanced stage disease (stage 3 and 4) as depicted in Figure 3.

Our study has certain limitations such as retrospective nature and small sample size. There is a need to gather more uniform population-based data and multicenter analysis before commenting on the guidelines and determining prognostic indicators.

## Conclusions

RMT squamous cell carcinoma is a rare entity with late presentation and usually in advanced stage results in poor outcome. These tumors have high risk of occult metastasis so elective neck dissection is mandatory for regional control. Close and involved surgical margins are related to local failure and adverse survival. Surgery followed by radiotherapy has shown improved overall three-year survival when compared with other patterns of treatment.
